# Effects of Postnatal Stress on the Development of Type 1 Diabetes in Bank Voles (*Clethrionomys glareolus*)

**DOI:** 10.1080/15438600303726

**Published:** 2003

**Authors:** Tonny Freimanis, Knud E. Heller, Bryan Schønecker, Mogens Bildsøe

**Affiliations:** 1 Zoological Institute University of Copenhagen Tagensvej 16 Copenhagen N. DK-2200 Denmark

## Abstract

Wild bank voles (*Clethrionomys glareolus*) kept in the laboratory under barren housing conditions develop high incidences of type 1 diabetes mellitus due to beta cell– specific lysis in association with the appearance of GAD65, IA-2, and insulin autoantibodies. Wild-caught and immediately analyzed voles show no histological signs of diabetes, and the disease may therefore be induced by circumstances related to the housing of the animals in captivity. We tested the possibility that postnatal stress by either maternal separation or water immersion at different intervals would induce diabetes in adult bank voles. We found that low-frequent stress during the first 21 days of life increases, whereas high-frequent stress markedly reduces, the incidence of type 1 diabetes in adulthood. These results differentiate the role of early-experienced stress on subsequent type 1 diabetes development and emphasize that the bank vole may serve as a useful new animal model for the disease.


*Experimental Diab. Res.*, 4:21–25, 2003
Copyright © 2003 Taylor & Francis
1543-8600/03 $12.00 + .00
DOI: 10.1080/15438600390214590

Taylor&Francis healthsciences

### Effects of Postnatal Stress on the Development of Type 1 Diabetes in Bank Voles (*Clethrionomys glareolus*)

**Tonny Freimanis, Knud E. Heller, Bryan Schønecker, and Mogens Bildsøe**

*Zoological Institute, University of Copenhagen, Copenhagen, Denmark*

**Wild bank voles (*Clethrionomys glareolus*) kept in the laboratory under barren housing conditions develop high incidences of type 1 diabetes mellitus due to beta cell–specific lysis in association with the appearance of GAD65, IA-2, and insulin autoantibodies. Wild-caught and immediately analyzed voles show no histological signs of diabetes, and the disease may therefore be induced by circumstances related to the housing of the animals in captivity. We tested the possibility that postnatal stress by either maternal separation or water immersion at different intervals would induce diabetes in adult bank voles. We found that low-frequent stress during the first 21 days of life increases, whereas high-frequent stress markedly reduces, the incidence of type 1 diabetes in adulthood. These results differentiate the role of early-experienced stress on subsequent type 1 diabetes development and emphasize that the bank vole may serve as a useful new animal model for the disease.**

**Keywords** Bank Voles; Postnatal Stress; Type 1 Diabetes

In recent studies focusing on the development of stereotypic behavior under barren and isolated housing conditions in wild-caught bank voles (*Clethrionomys glareolus*) (Parental [P]: n = 92) and their laboratory-bred offspring (F1: n = 248; F2: n = 270), we found that approximately 20% of the nonstereotyping voles in each of the three generations developed polydipsia (>21 mL/day water intake versus normally 12 mL/day) when approximately 2 months old [1, 2]. Polydipsia was more frequent among males (34%) than females (13%), but associated with weight loss in both sexes. When examining polydipsic (n = 20) and nonpolydipsic voles (n = 20), we found that polydipsia was associated with hyperglycemia (27.7 mM blood glucose versus 5.6 mM) and hyperlipidemia (42% versus 0%), strongly indicating that the polydipsic voles suffered from diabetes mellitus (unpublished data).

Subsequent studies [3] revealed that polydipsic but not nonpolydipsic voles showed almost complete loss of insulin-positive cells. Moreover, it was found that the beta cell–specific destruction in the pancreatic islets of polydipsic voles was associated with the presence of antibodies to the GAD65, IA-2, and insulin islet cell antigens.

Wild-caught and immediately analyzed voles showed no signs of pancreatic damages [3]. The disease may therefore be induced by circumstances associated with keeping the animals in captivity. One possibility is that housing voles isolated under barren conditions in the laboratory represents a stressful experience, which induces diabetes in the animals. Diabetes and development of polydipsia have been observed as a result of diabetes mellitus after glucocorticoid treatment, exposure to toxins, viral infections, surgical lesions, and other presumably stressful exposures [4–8]. Moreover, glucocorticoid hormones have been demonstrated to enhance the expression of glutamic acid decarboxylase (GAD) in nonobese diabetic (NOD) mouse beta cells and to increase the development of diabetes in the animal [9].

In the present study, we tested if early-experienced stress by maternal separation or water immersion at different intervals may have effects on subsequent development of type 1

---

Received 18 December 2002; accepted 9 January 2003.
This study was supported in part by The Danish Natural Science Research Council. The authors thank Åke Lernmark for helpful comments on an earlier version of this paper.
Address correspondence to Knud E. Heller, Zoological Institute, University of Copenhagen, Tagensvej 16, DK-2200 Copenhagen N., Denmark. E-mail: keheller@zi.ku.dk diabetes in the bank voles. Postnatal stress, including maternal deprivation, produces long-lasting and primarily enhancing modifications of the physiological stress response and stress-related anxiety behavior in common laboratory rodents [10–13]. Evidence is provided that the early stress effects culminate at the age of 3 months in rats [14], and that peripubertal environmental enrichment may lead to functional reversal of the changes [15].

### METHODS

### Animals and Treatment

Wild bank voles were trapped from May to November in a forest habitat on the island of Zealand, Denmark. In different trapping sessions, 90 traditional live traps were set and inspected twice a day. A total of 92 adult voles were trapped and immediately transferred to the laboratory for individually housing in small barren cages of transparent plastic (14 × 16 × 23 cm) under 12-hour light conditions (0800 to 2000). The cages were supplied with a woodcutting bed, and food (standard rat chow) and water were available ad libitum. Cage cleaning was performed every 2nd week or when necessary. A handful of grain mixture (pigeon blend) was given when the cages were cleaned. After at least 5 weeks in the laboratory, single mating pairs were transferred to larger enriched cages (15 × 22 × 38 cm) supplied with a woodcutting bed, toilet paper, and paper rolls. The breeding pairs were otherwise kept under conditions as described above. To avoid postpartum matings, males were removed from the females after 2 weeks and returned to isolation in their home cages. Among the resultant offspring (F1), 153 voles (88 males and 65 females) were randomly selected and divided into 3 groups assigned for the following experimental treatments: (a) 64 voles (33 males and 31 females) served as controls and were left undisturbed with their mothers and littermates; (b) 41 voles (23 males and 18 females) were assigned to maternal separation on postnatal days 7, 14, and 21 (×3); and (c) 48 voles (32 males and 16 females) were immersed in water on postnatal days 7, 14, and 21 (×3). An additional generation (F2) consisting of a total of 103 voles (56 males and 47 females) was established and randomly assigned to the following treatments; (d) 18 voles (10 males and 8 females) served as controls and were left undisturbed with their mothers and littermates; (e) 37 voles (20 males and 17 females) were subjected to separation on postnatal days 1 to 21 (×21); and (f) 48 voles (26 males and 22 females) were water-immersed on postnatal days 1 to 21 (×21).

Containers for separation and water immersion were alu-coated cartons (7 × 7 × 20 cm) supplied with either 1 cm woodcutting bed for separation or 15 cm lukewarm water for immersion. Each experimental treatment was performed by removing an entire litter from the breeding cage and placing it into a container where they stayed for either 4 hours (maternal separation) or 10 seconds (water immersion including swimming for older voles). All experimental voles were weaned at the age of 21 days and transferred to individual housing in plastic cages (14 × 16 × 23 cm) only supplied with woodcutting beds and otherwise treated as their isolated parents. Diabetes development was followed by measuring water intake (>21 mL/day for at least 1 month) in all voles until the age of 180 days.

### Statistical Analysis

We modeled risk of diabetes development by generalized linear modeling. The model included diabetes status at 180 days of age as response variable (2 levels), gender (2 levels), type of stressor (3 levels including controls), and frequency of stress treatments (2 levels) as predictors. Parameters were inferred by likelihood estimates using logits as link function and the binomial distribution. By stepwise estimation of all possible subsets of the full model, we chose the one taking the lowest Akaikes Information Criterion Value (AIC) (AIC = 281.9) against the full model (AIC = 289.9). This model was of the general form $P(x)$ = gender + frequency of stress treatment + type × frequency of stress treatment, and did not deviate significantly from the full model (Likelihood Ratio (LR) test: $X^2 = 5.98$, df = 7, $P = .542$). The Goodness of Fit

**TABLE 1**
Postnatal stress effects on adult diabetes development

| Bank voles | N | Male/female ratio | Diabetics n (%) | Male/female ratio (% diabetics) |
|---|---|---|---|---|
| Controls | 82 | 43/39 | 22 (27%) | 17/5 (40%/13%) |
| Separation ×3 | 41 | 23/18 | 15 (36%) | 12/3 (50%/17%) |
| Water immersion ×3 | 48 | 32/16 | 26 (53%) | 18/8 (56%/50%) |
| Separation ×21 | 37 | 20/17 | 6 (16%) | 5/1 (25%/6%) |
| Water immersion ×21 | 48 | 26/22 | 6 (13%) | 5/1 (19%/5%) |

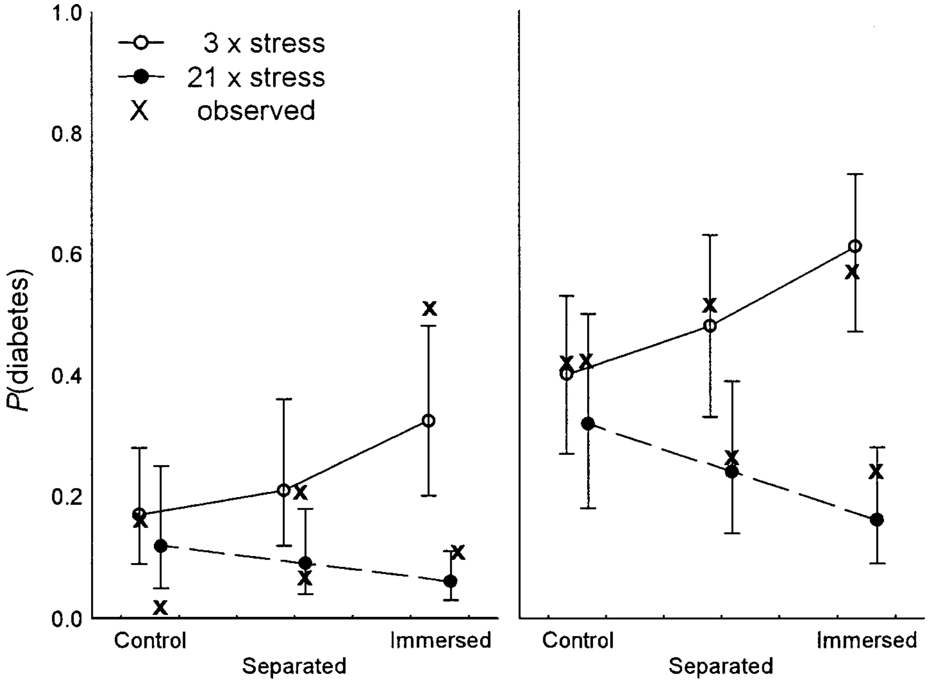

(*a*) (*b*)

**FIGURE 1**

Predicted and observed fractions of type 1 diabetes according to type and frequency of stress treatment in female (*a*) and male (*b*) bank voles. Bars indicate 95% confidence limits of predicted fractions.

(GOF) test failed to reveal significant deviations of predicted values from observations ($X^2 = 2.6$, df = 11, $P = .995$).

### RESULTS

When applying generalized linear modelling on our data (Table 1), we found significant effects of gender (LR test: df = 1, $X^2 = 15$, $P < .001$), stress frequency (LR test: df = 1, $X^2 = 13.6$, $P < .001$), and significant interaction between type and frequency of stress treatment (LR test: df = 2, $X^2 = 6.3$, $P = .044$), whereas there was no significant interaction between gender and type or frequency of stress treatment. Hence, although males and females deviated significantly in overall development of diabetes, they were equally affected by the stress treatments. These results are illustrated in Figure 1, which shows predicted and observed fractions of diabetes according to type and frequency of stress treatment. Because males and females were equally affected by the various stress treatments, we combined data from males and females in estimates of predicted odds ratios for each type and frequency of stress treatment against control treatment. Predicted odds ratios and their 95% confidence limits are shown in Figure 2. Confidence limits can be used tentatively as indications of significance between groups. The model clearly shows a bimodal mode of action of early stress treatment, indicating that stress factors were important to the appearance of type 1 diabetes in the voles. There were no

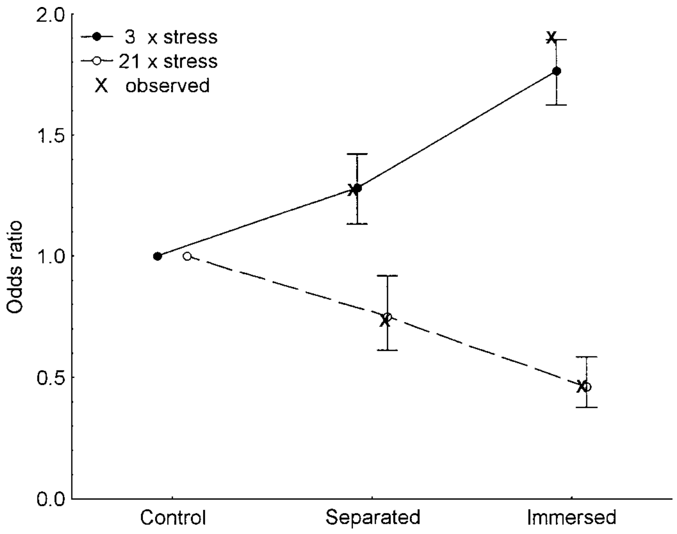

**FIGURE 2**

Predicted and observed odds ratios according to type and frequency of stress treatment against control treatment. Symbols as in Figure 1.

significant differences between the groups with respect to age at onset of diabetes (59 to 71 days).

### DISCUSSION

Provided that the high-frequent treatments performed here actually are experienced as more stressful than low-frequent treatments, the present results resemble previous findings in NOD mice [16–18]. Long-term repeated experimental stress delays the appearance of, decreases, or both, the incidences of diabetes in young NOD mice, whereas prenatal stress may accelerate the onset and increase the prevalence of diabetes in NOD females [17]. It has been suggested that the stress effects on type 1 diabetes in NOD mice are mediated at least partially by hormonal changes acting in a complex manner at different levels: the immune system, the islets of Langerhans, and other structures involved in glucose homeostasis [16]. The difference between male and female NOD mice was pronounced, however, in that low-frequent stress primarily accelerated diabetes in females whereas high-frequent stress reduced diabetes by about 50% in both males and females.

Early life stress plays an essential role in the development of adult hypothalamo-pituitary-adrenocortical responses to stress, but the effects seem to depend on the severity of the early-experienced stress. High-frequent postnatal stress leads to increased adrenocortical responses to stressful events in the adulthood, whereas low-frequent postnatal stress has the opposite effect [19–21]. These differential consequences of early stress might very well influence adult immune functioning in that high-level adrenocortical responses have been shown to suppress immunity, whereas low-level adrenocortical responses act in the opposite direction and are immunoenhancing [22]. We suggest that postnatal stress affects the prevalence of type 1 diabetes in adult voles through adrenocortical mediated effects on immune functioning, and that the direction of the effects depends on the frequency of early experienced stress. This assumption has of course to be supported by further studies of early stress effects on adrenocortical and immune functioning with special emphasis on processes of relevance to type 1 diabetes. Thus it cannot be ruled out that the voles subjected to high-frequent treatments in the present study may habituate to the treatments, and that high-frequent treatments therefore might be experienced as less stressful than low-frequent treatments. Because we have demonstrated that bank voles trapped in the wild and immediately analyzed most likely do not have diabetes but develop the disease in captivity in association with the appearance of type 1 diabetes GAD65 and IA-2 autoantibody markers [3], our present findings illustrate that imposing postnatal stress at various paradigms could be a useful tool for studying mechanisms of more general stress effects on type 1 diabetes. We also find that our study demonstrates the possibility of manipulating the prevalence of type 1 diabetes in bank voles and thereby underlines the potentials of using type 1 diabetic bank voles as a useful animal model for the disease in humans.

Spontaneous diabetes has previously been demonstrated in several rodent species [23–26] and more recently in the commonly used NOD mouse [27] and BB and KDP rats [28, 29]. Diabetic bank voles differ in several aspects from these other diabetic rodents. The most important ones appear to be the presence of significant autoantibody markers [3], higher incidence in males, early appearance, and longer survival time after onset of the disease [1].

### REFERENCES

[1] Schoenecker, B., Heller, K. E., and Freimanis, T. (2000) Development of stereotypies and polydipsia in wild caught bank voles (*Clethrionomys glareolus*) and their laboratory-bred offspring. Is polydipsia a symptom of diabetes mellitus? *Appl. Anim. Behav. Sci.,* **68,** 349–357.

[2] Schoenecker, B., and Heller, K. E. (2000) Indication of a genetic basis of stereotypies in laboratory-bred bank voles (*Clethrionomys glareolus*). *Appl. Anim. Behav. Sci.,* **68,** 339–347.

[3] Niklasson, B., Heller, K. E., Schønecker, B., Bildsøe, M., Daniels, T., Hampe, C. S., Widlund, P., Simonson, W. T., Schaefer, J. B., Rutledge, E., Bekris, L., Hammerle, L. P., Lindberg, A. M., Johansson, S., Örtqvist, E., Persson, B., and Lernmark, Å. (2002) Development of type 1 diabetes in wild bank voles associated with islet antibodies and the novel Ljungan virus. *Experimental Diab. Res.,* **4,** 35–44.

[4] Craighead, J. E. (1975) Animal models: Mice infected with the M variant of encephalomyocarditis virus. *Am. J. Pathol.,* **78,** 537–540.

[5] Hunt, C. E., Lindsey, J. R., and Walkley, S. U. (1976) Animal models of diabetes and obesity, including the PBB/Ld mouse. *Fed. Proc.,* **35,** 1206–1217.

[6] Mormedes, J. P., and Rossini, A. A. (1981) Animal models of diabetes. *Am. J. Med.,* **70,** 353–360.

[7] Schlosser, M. J., Kapeghian, J. C., and Verlangieri, A. J. (1984) Effects of streptozotocin in the male guinea pig: A potential animal model for studying diabetes. *Life Sci.,* **35,** 649–655.

[8] Tarui, S., Yamada, K., and Hanafusa, T. (1987) Animal models utilized in the research of diabetes mellitus with special reference to insulitis-associated diabetes. *Prog. Clin. Biol., Res.,* **229,** 211–223.

[9] Kim, K. S., Kang, Y., Choi, S. E., Kim, H. M., Sun, B., Jun, H. S., and Yoon, J. W. (2002) Modulation of glucocorticoid-induced GAD expression in pancreatic beta-cells by transcriptional activation of the GAD67 promoter and its possible effect on the development of diabetes. *Diabetes,* **51,** 2764–2772.

[10] Penke, Z., Felszeghy, K., Fernette, B., Sage, D., Nyakas, C., and Burlet, A. (2001) Postnatal maternal deprivation produces long-lasting modifications of the stress response, feeding and stress-related behavior in the rat. *Eur. J. Neurosci.,* **14,** 747–755.

[11] Schmidt, M., Oitzl, M. S., Levine, S., and de Kloet, E. R. (2002) The HPA system during the postnatal evelopment of CD1 mice and the effects of maternal deprivation. *Brain Res. Dev. Brain Res.,* **15,** 39–49.

[12] Vazquez, D. M., Eskandari, R., Zimmer, C. A., Levine, S., and Lopez, J. F. (2002) Brain 5-HT receptor system in the stressed infant rat: Implications for vulnerability to substance abuse. *Psychoneuroendocrinology,* **27,** 245–272.

[13] Zhang, L. X., Levine, S., Dent, G., Zhan, Y., Xing, G., Okimoto, D., Kathleen Gordon, M., Post, R. M., and Smith, M. A. (2002) Maternal deprivation increases cell death in the infant rat brain. *Brain Res. Dev. Brain. Res.,* **31,** 1–11.

[14] Workel, J. O., Oitzl, M. S., Flutter, M., Lessher, H., Karssen, A., and de Kloet, E. R. (2001) Differential and age-dependent effects of maternal deprivation on the hypothalamic-pituitary-adrenal axis of brown Norway rats from youth to senescence. *J. Neuroendocrinol.,* **13,** 569–580.

[15] Francis, D. D., Diorio, J., Plotsky, P. M., and Meaney, M. J. (2002) Environmental enrichment reverses the effects of maternal separation on stress reactivity. *J. Neurosci.,* **22,** 7840–7843.

[16] Durant, S., Coulaud, J., Amrani, A., el Hasnaoui, A., Dardenne, M., and Homo-Delarche, F. (1993) Effects of various environmental stress paradigms and adrenalectomy on the expression of autoimmune type 1 diabetes in the non-obese diabetic (NOD) mouse. *J. Autoimmun.,* **6,** 735–751.

[17] Saravia-Fernandez, F., Durant, S., el Hasnaoui, A., Dardenne, M., and Homo-Delarche, F. (1996) Environmental and experimental procedures leading to variations in the incidence of diabetes in the nonobese diabetic (NOD) mouse. *Autoimmunity,* **24,** 113–121.

[18] Durant, S., Christeff, N., Coulaud, J., Ninez, E. E., Dardenne, M., and Homo-Delarche, F. (1998) Basal concentrations of various steroids in the nonobese diabetic (NOD) mouse and effect of immobilization stress. *Autoimmunity,* **28,** 249–258.

[19] Cook, C. J. (1999) Patterns of weaning and adult response to stress. *Physiol. Behav.,* **67,** 803–808.

[20] Abraham, I. M., and Kovacs, K. J. (2000) Postnatal handling alters the activation of stress-related neuronal circuits. *Eur. J. Neurosci.,* **12,** 3003–3014.

[21] Gilad, V. H., Rabey, J. M., Eliyayev, Y., and Gilad, G. M. (2000) Different effects of acute postnatal stressors and long-term postnatal handling on stress-induced changes in behavior and in ornithine decarboxylase activity in adult rats. *Dev. Brain Rec.,* **120,** 255–259.

[22] Dhabhar, F. S., and McEven, B. S. (1999) Enhancing versus suppressive effects of stress hormones on skin immune function. *Proc. Natl. Acad. Sci. U.S.A.,* **96,** 1059–1064.

[23] Meir, H. (1960) Diabetes mellitus in animals: A review. *Diabetes,* **9,** 485–489.

[24] Schmidt-Nielsen, K., Haines, H. B., and Hackel, D. E. (1964) Diabetes mellitus in the sand rat induced by standard laboratory diets. *Science,* **143,** 689–690.

[25] Gonet, A. E., Mougin, J., and Renold, A. E. (1965) Hyperplasia and hypertrophy of the islets of Langerhans, obesity and diabetes in the mouse (*Acomys dimidiatus*). *Acta Endocrinol. Suppl.,* **100,** 135.

[26] Wise, P. H., Weir, B. J., Hime, J. M., and Forrest, E. (1972) The diabetic syndrome in the Tuco-Tuco (*Ctenomys talarum*). *Diabetologica,* **8,** 165–172.

[27] Delovich, T. L., and Singh, B. (1997) The nonobese diabetic mouse as a model of autoimmune diabetes: Immune dysregulation gets the NOD. *Immunity,* **7,** 727–738.

[28] Crisa, L., Mordes, J. P., and Rossini, A. A. (1992) Autoimmune diabetes mellitus in the BB rat. *Diabetes Metab. Rev.,* **8,** 4–37.

[29] Kawano, K., Hirashima, T., Mori, S., Saitoh, Y., Kurosumi, M., and Natori, T. (1991) New inbred strain of Long-Evans Tokushima lean rats with IDDM without lymphopenia. *Diabetes,* **40,** 1375–1381.

